# Complete genome sequence of *Syntrophothermus lipocalidus* type strain (TGB-C1^T^)

**DOI:** 10.4056/sigs.1233249

**Published:** 2010-12-15

**Authors:** Olivier Duplex Ngatchou Djao, Xiaojing Zhang, Susan Lucas, Alla Lapidus, Tijana Glavina Del Rio, Matt Nolan, Hope Tice, Jan-Fang Cheng, Cliff Han, Roxanne Tapia, Lynne Goodwin, Sam Pitluck, Konstantinos Liolios, Natalia Ivanova, Konstantinos Mavromatis, Natalia Mikhailova, Galina Ovchinnikova, Amrita Pati, Evelyne Brambilla, Amy Chen, Krishna Palaniappan, Miriam Land, Loren Hauser, Yun-Juan Chang, Cynthia D. Jeffries, Manfred Rohde, Johannes Sikorski, Stefan Spring, Markus Göker, John C. Detter, Tanja Woyke, James Bristow, Jonathan A. Eisen, Victor Markowitz, Philip Hugenholtz, Nikos C. Kyrpides, Hans-Peter Klenk

**Affiliations:** 1 HZI – Helmholtz Centre for Infection Research, Braunschweig, Germany; 2 Los Alamos National Laboratory, Bioscience Division, Los Alamos, New Mexico, USA; 3 DOE Joint Genome Institute, Walnut Creek, California, USA; 4 DSMZ - German Collection of Microorganisms and Cell Cultures GmbH, Braunschweig, Germany; 5 Biological Data Management and Technology Center, Lawrence Berkeley National Laboratory, Berkeley, California, USA; 6 Oak Ridge National Laboratory, Oak Ridge, Tennessee, USA; 7 University of California Davis Genome Center, Davis, California, USA

**Keywords:** anaerobic, motile, Gram-negative, syntrophism with methanogen, crotonate, butyrate, isobutyrate, *Syntrophomonadaceae*, GEBA

## Abstract

*Syntrophothermus lipocalidus* Sekiguchi *et al*. 2000 is the type species of the genus *Syntrophothermus*. The species is of interest because of its strictly anaerobic lifestyle, its participation in the primary step of the degradation of organic maters, and for releasing products which serve as substrates for other microorganisms. It also contributes significantly to maintain a regular pH in its environment by removing the fatty acids through β-oxidation. The strain is able to metabolize isobutyrate and butyrate, which are the substrate and the product of degradation of the substrate, respectively. This is the first complete genome sequence of a member of the genus *Syntrophothermus* and the second in the family *Syntrophomonadaceae*. Here we describe the features of this organism, together with the complete genome sequence and annotation. The 2,405,559 bp long genome with its 2,385 protein-coding and 55 RNA genes is a part of the *** G****enomic* *** E****ncyclopedia of* *** B****acteria and* *** A****rchaea * project.

## Introduction

Strain TGB-C1^T^ (= DSM 12680) is the type strain of *Syntrophothermus lipocalidus* [1] which in turn is the type species of the genus *Syntrophothermus* [2]. Currently, this is the only species placed in the genus *Syntrophothermus*. The genus name derives from the Greek words “*syn*”, together with, “*trophos*”, one who feeds, and “*thermus*”, hot, referring to a thermophilic bacterium growing in syntrophic association with hydrogenotrophic organisms at high temperature of around 55°C [1]. The species epithet derives from the Greek word “*lipos*”, fat, and from the Latin adjective “*calidus*”, expert, referring to the organisms trait of specifically utilizing fatty acids [1]. Strain TGB-C1^T^ was isolated from granular sludge in a thermophilic upflow anaerobic sludge blanket (UASB) [1]. No further cultivated strains belonging to the species *S. lipocalidus* have been described so far. Here we describe the features of this organism, together with the complete genome sequence and annotation.

## Classification and features

**Figure 1:**
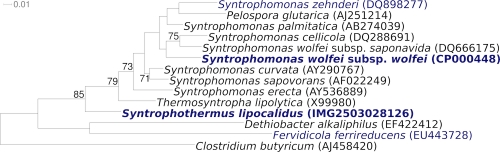
Phylogenetic tree highlighting the position of *S. lipocalidus* TGB-C1^T^ relative to the type strains within the family *Syntrophomonadaceae*. The trees were inferred from 1,434 aligned characters [3,4] of the 16S rRNA gene sequence under the maximum likelihood criterion [5] and rooted in accordance with the current taxonomy [6]. The branches are scaled in terms of the expected number of substitutions per site. Numbers above branches are support values from 1,000 bootstrap replicates [7] if larger than 60%. Lineages with type strain genome sequencing projects registered in GOLD [8] are shown in blue, published genomes in bold [9].

The 16S rRNA gene sequence of strain TGB-C1^T^ revealed an only distant relationship with the other representatives of the family *Syntrophomonadaceae* [1] (Figure1), with *Thermosyntropha lipolytica* [10] showing the highest degree of sequence similarity (88.1%). The sequence distances of strain TGB-C1^T^ to other members of this family were 13.6% with *Syntrophomonas wolfei* subsp. *wolfei*, 14.0% with *S. bryantii,* and 14.8% with *S. sapovorans*, respectively [1]. Further analysis showed 98% 16S rRNA gene sequence identity with an uncultured bacterium represented by clone AR80B63 (AB539943) from the high-temperature Yabase oil field in Japan. The sequence of the 16S rRNA gene of strain TGB-C1^T^ is identical with two unclassified sequences from an hydrothermal vent metagenome LCHCB.C3615 [11] and from human gut metagenome DNA (contig sequence: F2-Y_011332) [12] (status August 2010), indicating that members of the species, genus and even family are widely represented in the habitats screened so far.

A representative genomic 16S rRNA sequence of *S. lipocalidus* TGB-C1^T^ was also compared using BLAST with the most resent release of the Greengenes database [13] and the relative frequencies of taxa and keywords, weighted by BLAST scores, were determined. The five most frequent genera were *Moorella* (44.1%), *Syntrophomonas* (33.8%), *Clostridium* (6.0%), *Syntrophothermus* (5.6%) and *Carboxydocella* (3.5%). The species yielding the highest score was *Moorella thermoautotrophica*. The five most frequent keywords within the labels of environmental samples which yielded hits were 'microbial' (5.5%), 'anaerobic' (4.2%), 'rice' (2.9%), 'soil' (2.8%) and 'populations' (2.8%). The three most frequent keywords within the labels of environmental samples which yielded hits of a higher score than the highest scoring species were 'temperature' (8.2%), 'acetate, coupled, evidence, field, hydrogenotrophic, methanogenesis, oil, oxidation, petroleum, reservoir, syntrophic, yabase' (5.0%) and 'dependent, hot, muddy, reducing, sediment, southwestern, spring, succession, sulfate, taiwan' (3.2%). These keywords largely fit to what is known about the ecology and physiology of strain TGB-C1^T^ [1].

Figure 1 shows the phylogenetic neighborhood of *S. lipocalidus* TGB-C1^T^, in a 16S rRNA based tree. The sequences of the two 16S rRNA gene copies in the genome differ from each other by up to two nucleotides, and differ by up to two nucleotides from the previously published 16S rRNA sequence (AB021305).

**Figure 2:**
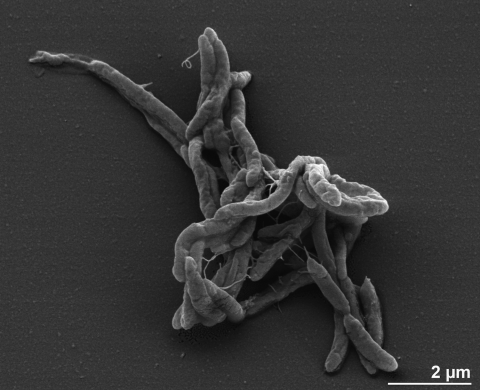
Scanning electron micrograph of *S. lipocalidus* TGB-C1^T^

Cells of strain TGB-C1^T^ are Gram-negative, slightly curved rods with round ends and weakly motile with flagella, 2.4 - 4.0 µm long and 0.4 - 0.5 µm wide (Figure 2 and Table 1) [1], occurring singly or in pairs. Roll-tube isolation revealed the presence of small white colonies, lens-shaped and 0.1 - 0.2 mm in diameter [1]. The growth rate of the strain TGB-C1^T^ on 10 mM crotonate was 0.93 ± 0.01 d^-1^. Strain TGB-C1^T^ is strictly anaerobic [1]. It grows on crotonate at temperatures between 45°C and 60°C, with the optimum at 55°C. The pH_25°C_ range for growth is 5.8-7.5, with an optimum at 6.5-7.0 [1]. Strain TGB-C1^T^ metabolizes in two ways, in pure culture only in the presence of the unsaturated fatty acid crotonate and in co-culture with *Methanobacterium thermoautotrophicum* strain ΔH in the presence of saturated fatty acids [1]. In pure culture, the fermentation products are acetate and butyrate in equimolar amounts. In co-culture with *M. thermoautotrophicum*, the substrates used are butyrate, straight-chain fatty acids from C_4_ to C_10_ and isobutyrate [1]. By oxidizing fatty acids, *S. lipocalidus* produces acetate and hydrogen [1], the latter of which is then scavenged by the syntrophic methanogen *M. thermoautotrophicum* [1]. Syntrophic hydrogenotrophic interactions with bacteria from the genus *Methanobacterium* have been also observed in the genome sequenced bacterium *Aminobacterium colombiense* strain ALA-1^T^ from the phylum *Synergistetes* [26]. *S. lipocalidus* is the only species in the family *Syntrophomonadaceae* that is able to metabolize isobutyrate [2]. Neither yeast extract nor tryptone significantly stimulates growth [1]. In the presence of butyrate as electron donor, the following compounds do not serve as electron acceptors: sulfate, nitrate, sulfite, thiosulfate, fumarate, Fe(III)-nitrilotriacetate [1]. Cell growth is inhibited by ampicillin, chloramphenicol, kanamycin, neomycin, rifampin or vancomycin (each 50 µg ml^-1^) [1].

**Table 1:** Classification and general features of *S. lipocalidus* TGB-C1^T^ in according with the MIGS recommendations [14]

**MIGS ID**	**Property**	**Term**	**Evidence code**
	Current classification	Domain *Bacteria*	TAS [15]
		Phylum *Firmicutes*	TAS [16,17]
		Class *Clostridia*	TAS [18,19]
		Order *Clostridiales*	TAS [20,21]
		Family *Syntrophomonadaceae*	TAS [22,23]
		Genus *Syntrophothermus*	TAS [1]
		Species *Syntrophothermus lipocalidus*	TAS [1]
		Type strain TGB-C1	TAS [1]
	Gram stain	negative	TAS [1]
	Cell shape	slightly curved rods with round ends	TAS [1]
	Motility	weakly motile by flagella	TAS [1]
	Sporulation	None	TAS [1]
	Temperature range	45°C–60°C	TAS [1]
	Optimum temperature	55°C	TAS [1]
	Salinity	< 0.5% NaCl	TAS [1]
MIGS-22	Oxygen requirement	obligately anaerobic	TAS [1]
	Carbon source	crotonate in pure culture; fatty acids with 4-10 carbon atoms including isobutyrate in syntrophy	TAS [1]
	Energy source	crotonate	TAS [1]
MIGS-6	Habitat	not reported	NAS
MIGS-15	Biotic relationship	syntrophic with methanogens	NAS
MIGS-14	Pathogenicity	not reported	NAS
	Biosafety level	1	TAS [24]
	Isolation	granular sludge in a thermophilic upflow anaerobic sludge blanket (UASB) reactor	TAS [1]
MIGS-4	Geographic location	most probably Japan	TAS [1]
MIGS-5	Sample collection time	2000 or before	TAS [1]
MIGS-4.1 MIGS-4.2	Latitude Longitude	not reported	NAS
MIGS-4.3	Depth	not reported	NAS
MIGS-4.4	Altitude	not reported	NAS

Evidence codes - IDA: Inferred from Direct Assay (first time in publication); TAS: Traceable Author Statement (i.e., a direct report exists in the literature); NAS: Non-traceable Author Statement (i.e., not directly observed for the living, isolated sample, but based on a generally accepted property for the species, or anecdotal evidence). These evidence codes are from of the Gene Ontology project [25]. If the evidence code is IDA, then the property was directly observed by one of the authors or an expert mentioned in the acknowledgements.

### Chemotaxonomy

To date, no experimental reports have specified the lipid composition of the cell envelope of strain TGB-C1^T^. Nevertheless, the cell envelope of the strain TGB-C1^T^ was Gram-negative stained, although electron micrographs and the 16S rRNA analysis showed that the strain was affiliated to the Gram-positive bacteria [1]. This feature was also observed for another member of the family *Syntrophomonadaceae*, *S. bryantii* [22,27]. The cell envelope is composed of the cytoplasmic membrane, an electron-dense layer, which is most probably made of peptidoglycan, and an electron-dense outermost wall [1].

## Genome sequencing and annotation

### Genome project history

This organism was selected for sequencing on the basis of its phylogenetic position [28], and is part of the *** G****enomic* *** E****ncyclopedia of* *** B****acteria and* *** A****rchaea * project [29]. The genome project is deposited in the Genome OnLine Database [8] and the complete genome sequence is deposited in GenBank. Sequencing, finishing and annotation were performed by the DOE Joint Genome Institute (JGI). A summary of the project information is shown in Table 2.

**Table 2:** Genome sequencing project information

**MIGS ID**	**Property**	**Term**
MIGS-31	Finishing quality	Finished
MIGS-28	Libraries used	Three genomic libraries: 454 pyrosequence standard library and; paired end library (10.2 kb insert size); Illumina standard library
MIGS-29	Sequencing platforms	454 GS FLX Titanium, Illumina GAii
MIGS-31.2	Sequencing coverage	103.3 × pyrosequence, 81.3 × Illumina
MIGS-30	Assemblers	Newbler version 2.1-PreRelease-4-28-2009, Velvet, phrap
MIGS-32	Gene calling method	Prodigal 1.4, GenePRIMP
	INSDC ID	CP002048
	Genbank Date of Release	June 7, 2010
	GOLD ID	Gc012392
	NCBI project ID	37873
	Database: IMG-GEBA	2502957035
MIGS-13	Source material identifier	DSM 12680
	Project relevance	Tree of Life, GEBA

### Growth conditions and DNA isolation

*S. lipocalidus* TGB-C1^T^, DSM 12680, was grown anaerobically in DSMZ medium 870 (*Syntrophothermus* medium) [30] at 55°C. DNA was isolated from 0.5-1 g of cell paste using the Jetflex Genomic DNA Purification kit (GENOMED 600100) following the standard protocol as recommended by the manufacturer, with 30 min incubation at 58°C for cell lysis.

### Genome sequencing and assembly

The genome was sequenced using a combination of Illumina and 454 sequencing platforms. All general aspects of library construction and sequencing can be found at the JGI website [31]. Pyrosequencing reads were assembled using the Newbler assembler version 2.1-PreRelease-4-28-2009-gcc-3.4.6-threads (Roche). The initial Newbler assembly consisting of 16 contigs in one scaffold was converted into a phrap assembly by making fake reads from the consensus, collecting the read pairs in the 454 paired end library. Illumina GAii sequencing data (704 Mb) was assembled with Velvet [32] and the consensus sequences were shredded into 1.5 kb overlapped fake reads and assembled together with the 454 data. 454 draft assembly was based on 248.9 Mb 454 draft data and all of the 454 paired end data. Newbler parameters are -consed -a 50 -l 350 -g -m -ml 20. The Phred/Phrap/Consed software package [33] was used for sequence assembly and quality assessment in the following finishing process. After the shotgun stage, reads were assembled with parallel phrap (High Performance Software, LLC). Possible mis-assemblies were corrected with gapResolution [31], Dupfinisher, or sequencing cloned bridging PCR fragments with subcloning or transposon bombing (Epicentre Biotechnologies, Madison, WI) [34]. Gaps between contigs were closed by editing in Consed, by PCR and by Bubble PCR primer walks (J.-F.Chang, unpublished). A total of 37 additional reactions were necessary to close gaps and to raise the quality of the finished sequence. Illumina reads were also used to correct potential base errors and increase consensus quality using a software Polisher developed at JGI [35]. The error rate of the completed genome sequence is less than 1 in 100,000. Together, the combination of the Illumina and 454 sequencing platforms provided 184.6 × coverage of the genome. Final assembly contains 815,143 pyrosequence and 5,434,428 Illumina reads.

### Genome annotation

Genes were identified using Prodigal [36] as part of the Oak Ridge National Laboratory genome annotation pipeline, followed by a round of manual curation using the JGI [37]. The predicted CDSs were translated and used to search the National Center for Biotechnology Information (NCBI) nonredundant database, UniProt, TIGRFam, Pfam, PRIAM, KEGG, COG, and InterPro databases. Additional gene prediction analysis and functional annotation was performed within the (IMG-ER) platform [38].

## Genome properties

**Figure 3:**
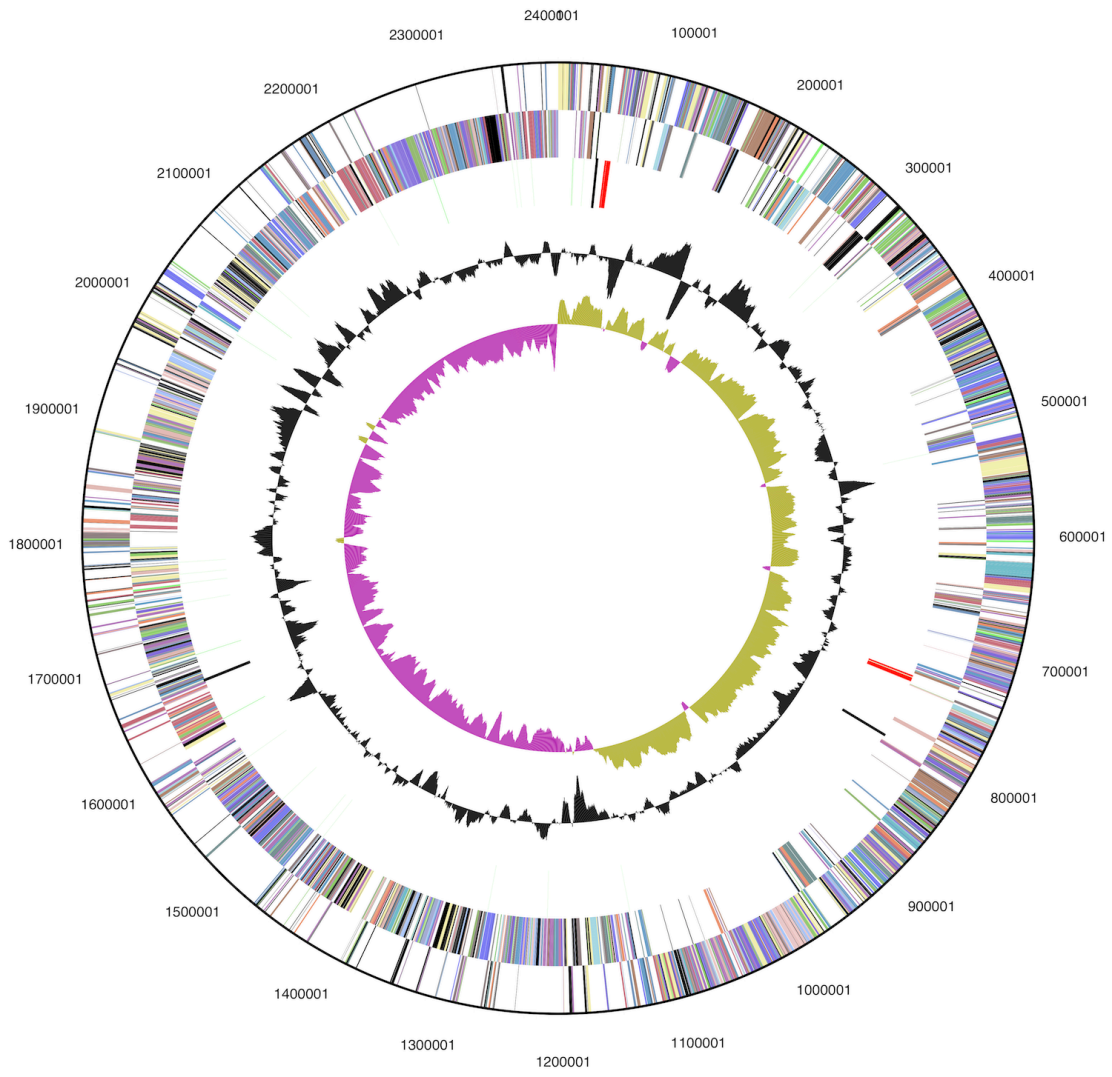
Graphical circular map of the genome. From outside to the center: Genes on forward strand (color by COG categories), Genes on reverse strand (color by COG categories), RNA genes (tRNAs green, rRNAs red, other RNAs black), GC content, GC skew.

The genome consists of a 2,405,559 bp long chromosome with a 51.0% GC content (Table 3 and Figure 3). Of the 2,440 genes predicted, 2,385 were protein-coding genes, and 55 RNAs; 72 pseudogenes were also identified. The majority of the protein-coding genes (70.7%) were assigned with a putative function while the remaining ones were annotated as hypothetical proteins. The distribution of genes into COGs functional categories is presented in Table 4.

**Table 3:** Genome Statistics

**Attribute**	**Value**	**% of Total**
Genome size (bp)	2,405,559	100.00%
DNA coding region (bp)	2,078,709	86.41%
DNA G+C content (bp)	1,226,580	50.99%
Number of replicons	1	
Extrachromosomal elements	0	
Total genes	2,440	100.00%
RNA genes	55	2.25%
rRNA operons	2	
Protein-coding genes	2,385	97.75%
Pseudo genes	72	2.95%
Genes with function prediction	1,726	70.74%
Genes in paralog clusters	348	14.26%
Genes assigned to COGs	1,767	72.42%
Genes assigned Pfam domains	1,912	78.26%
Genes with signal peptides	603	24.71%
Genes with transmembrane helices	545	22.34%
CRISPR repeats	2	

**Table 4:** Number of genes associated with the general COG functional categories

**Code**	**value**	**%age**	**Description**
J	144	7.4	Translation, ribosomal structure and biogenesis
A	0	0.0	RNA processing and modification
K	113	5.8	Transcription
L	123	6.3	Replication, recombination and repair
B	4	0.2	Chromatin structure and dynamics
D	32	1.6	Cell cycle control, cell division, chromosome partitioning
Y	0	0.0	Nuclear structure
V	33	1.7	Defense mechanisms
T	107	5.5	Signal transduction mechanisms
M	96	4.9	Cell wall/membrane/envelope biogenesis
N	81	4.1	Cell motility
Z	0	0.0	Cytoskeleton
W	0	0.0	Extracellular structures
U	66	3.4	Intracellular trafficking and secretion, and vesicular transport
O	74	3.8	Posttranslational modification, protein turnover, chaperones
C	144	7.4	Energy production and conversion
G	67	3.4	Carbohydrate transport and metabolism
E	144	7.4	Amino acid transport and metabolism
F	58	3.0	Nucleotide transport and metabolism
H	112	5.7	Coenzyme transport and metabolism
I	98	5.0	Lipid transport and metabolism
P	70	3.6	Inorganic ion transport and metabolism
Q	26	1.3	Secondary metabolites biosynthesis, transport and catabolism
R	205	10.5	General function prediction only
S	158	8.1	Function unknown
-	673	27.6	Not in COGs
